# Anthropometric traits and risk of multiple myeloma: differences by race, sex and diagnostic clinical features

**DOI:** 10.1038/s41416-024-02723-6

**Published:** 2024-06-07

**Authors:** Kevin D. Arnold, Krystle L. Ong, Gayathri Ravi, Hannah Cutshall, Kalyn Purnell, Meredith C. Wessel, Kelly N. Godby, Susan Bal, Smith Giri, Laura Q. Rogers, Wendy Demark-Wahnefried, Faith E. Davies, Luciano J. Costa, Gareth J. Morgan, Brenda M. Birmann, Elizabeth E. Brown

**Affiliations:** 1 Department of Pathology, Heersink School of Medicine, University of Alabama at Birmingham, Birmingham, AL 35294 USA; 2 Division of Hematology and Medical Oncology, Department of Medicine, Heersink School of Medicine, University of Alabama at Birmingham, Birmingham, AL 35294 USA; 3 O’Neal Comprehensive Cancer Center, University of Alabama at Birmingham, Birmingham, AL 35294 USA; 4 Department of Pathology, University of Arkansas for Medical Sciences, Little Rock, AR 72205 USA; 5 Division of Preventive Medicine, Department of Medicine, Heersink School of Medicine, University of Alabama at Birmingham, Birmingham, AL 35294 USA; 6 Department of Nutrition Sciences, School of Health Professions, University of Alabama at Birmingham, Birmingham, AL 35294 USA; 7 Perlmutter Comprehensive Cancer Center, Langone Medical Center, New York University, New York, NY 10021 USA; 8 Channing Division of Network Medicine, Department of Medicine, Brigham and Women’s Hospital and Harvard Medical School, Boston, MA 02115 USA

**Keywords:** Risk factors, Cancer epidemiology, Myeloma

## Abstract

**Background:**

Obesity is an established modifiable risk factor for multiple myeloma (MM). However, associations of obesity and MM risk in Black populations, for whom obesity and MM are more common, is less clear.

**Methods:**

Using participants enrolled in the Integrative Molecular And Genetic Epidemiology study, we evaluated the association of anthropometric traits with MM risk overall, stratified by race and sex. Among cases, we assessed the association of BMI with the presence of myeloma-defining events.

**Results:**

We observed an 18% increase in MM risk for every 5 kg/m^2^ increase in usual adult BMI. Participants with severe obesity (BMI ≥ 40 kg/m^2^) had the highest risk compared to those with a normal usual adult BMI (18.5–24.9 kg/m^2^; OR = 1.87, 95% CI 1.25–2.80), particularly among Black men (OR = 3.94, 95% CI 0.90–17.36). Furthermore, MM cases with overweight/obesity (BMI ≥ 25 kg/m^2^) were more likely to present at diagnosis with low renal function (OR = 1.62, 95% CI 1.09–2.40), deletion 13q (OR = 1.73, 95% CI 1.08–2.76) and lytic lesions or compression fractures (OR = 2.39, 95% CI 0.82–7.01) and less likely to present with severe diffuse osteopenia (OR = 0.51, 95% CI 0.31–0.81).

**Conclusions:**

Findings underscore the importance of obesity as a modifiable risk factor for MM, particularly in high-risk populations, and for the clinical presentation of disease.

## Background

Multiple myeloma (MM) is the second most common haematologic malignancy in the US with an estimated 35,730 new cases diagnosed in 2023, and it is the most common blood cancer affecting Black populations [1]. Although standardised incidence rates are increasing and are 2 to 3 times higher in Black than in White populations [2, 3], overall survival does not notably differ by race [4]. This suggests that underlying differences in driver events that accumulate early in the monoclonal evolution of terminally differentiated plasma cells provide a trajectory that predisposes Black persons to greater risk.

Risk factors for MM include increasing age, male sex, Black race, family history or presence of plasma cell dyscrasia and obesity [5]. Of these, obesity is the only known modifiable risk factor for MM [6–8] and its precursor, monoclonal gammopathy of undetermined significance (MGUS) [9–12]. Strong and consistent evidence supports positive associations between increasing MM risk with increasing body mass index (BMI) [13–17], excess BMI at different time points across the lifespan [18–21], medium to increased body size trajectory [22], and anthropometric measures of height [15, 23, 24], weight [23, 25] and waist or hip circumference [22, 25]. This body of evidence led to the International Agency for Research on Cancer to conclude that MM is an obesity-related cancer [26].

Although reducing excess body fat may mitigate MM risk, associations of obesity and MM risk among Black persons, for whom obesity and MM are more common, remains unclear. Previously published epidemiologic case-control [27] and cohort [16, 28] studies produced mixed results. However, definitions of obesity differed across studies. In this study, we characterised the excess risk of MM in Black adults associated with anthropometric traits and BMI including severe obesity, and the influence of obesity on the clinical presentation of MM, which has not been previously reported.

## Methods

### Study population

Among participants enrolled in the Integrative Molecular And Genetic Epidemiology (IMAGE) study [29], we evaluated the risk of MM associated with anthropometric traits including height, weight and BMI at diagnosis and 1 year before diagnosis stratified by self-reported race and sex. In addition among cases, we assessed associations of anthropometric traits with the presence of myeloma-defining events (MDE). Approvals by the Institutional Review Board were obtained prior to study initiation.

### Case definition

Eligible cases were self-reported Black and White patients recruited from the University of Alabama at Birmingham (UAB) Multidisciplinary Myeloma Clinic between 2009 and 2020. Patients with a MM diagnosis (International Classification of Diseases [ICD], 9th Revision codes 203.0, 203.1; ICD-10, Clinical Modification codes C90.0, C90.1) were identified and confirmed based on the International Multiple Myeloma Working Group classification for MM [30]. Each case was reviewed by an expert panel to minimise misclassification. Of the 1035 eligible MM cases identified, 850 (82.0%) were enrolled (76.2% Black; 86.8% White; 0.1% Asian). Consistent with the previous report [29], reasons for refusal to participate included declined interview (17.4% Black; 10.1% White), illness (1.2% Black; 1.7% White), or other (4.7% Black; 1.4% White). One self-reported Asian participant (*n* = 1) was excluded from this comparison of self-reported Black and White participants, leaving 849 confirmed MM cases for analysis.

### Control selection

Controls were sampled from a population-based database established and maintained by the UAB Survey Research Unit, which includes US Census and Centers for Disease Control population databases established from list-assisted random digit dialing methods [29]. Eligible controls were residents of the Southeast US, at least 21 years of age, without a self-reported history of plasma cell dyscrasia, other cancers excluding non-melanoma skin cancer, solid organ transplant or HIV-1 infection. One to two controls were randomly selected, and frequency matched to cases on self-reported race (Black, White), sex, age (±5 years) and geography. Among the 2079 eligible controls, 1405 (67.6%) were enrolled. Response rates for controls were 61.9% for Black and 74.6% for White participants. Declining to be interviewed was the most common reason for refusal (29.2% Black; 19.0% White), followed by other (5.9% Black; 4.1% White) and illness (3.0% Black; 2.30% White). Enrolled controls later discovered to be duplicates (*n* = 12), a blood relative of a case (*n* = 22), those who reported a shared residential environment with a case or other enrolled control for 2 or more years (*n* = 23), or who reported diagnoses of MGUS (*n* = 1), myelodysplastic syndrome (*n* = 6), HIV-1 infection or solid organ transplant (*n* = 14) were excluded to minimise the influence of environmental factors on BMI and MM associations.

### Anthropometric traits

Detailed information, including the social construct of race, anthropometric, socioeconomic and behavioural risk factors, was obtained by structured interview at enrollment. Self-reported racialized groups (Non-Hispanic Black, Non-Hispanic White; herein referred to as Black and White) were collected to enable an improved understanding of the disparity in MM incidence observed in those who self-identify as Black [2, 3]. In addition, we collected maximum adult height (cases and controls) and height at diagnosis (cases) and enrollment (controls), as well as weight at diagnosis and 1 year before diagnosis (cases) and current weight (controls). We defined sex-specific quartiles for each height and weight variable based on the corresponding distributions among controls (male current height [cm], Q1:152–173, Q2:174–178, Q3:179–183, Q4:>183; female current height [cm], Q1:137–160, Q2:161–165, Q3:166–168, Q4:>168; male maximum adult height [cm], Q1:152–175, Q2:176–180, Q3:181–183, Q4:>183; female maximum adult height [cm], Q1:140–160, Q2:161–165, Q3:166–170, Q4:>170; male current weight [kg], Q1:52.2–78.7, Q2:78.8–88.5, Q3:88.6–102.1, Q4:>102.1; and female current weight [kg], Q1:44–65.8, Q2:65.9–77.1, Q3:77.2–90.7, Q4:>90.7). We calculated BMI (kg/m^2^) at two time points: current BMI from height and weight at diagnosis for cases and height and weight at enrollment for controls; and usual adult BMI from maximum adult height and weight 1 year before diagnosis for cases and from maximum adult height and weight at enrollment for controls.

### Clinical measures

Among cases only, diagnostic MDE and clinical features were ascertained from medical history, and pathology, laboratory and radiology reports. Clinical features of interest included percent clonal bone marrow plasma cells, select laboratory measures: β2-microglobulin and lactate dehydrogenase; serum paraprotein assessments including total monoclonal protein, immunoglobulin (Ig) isotype, involved light chain for clonality, and involved:uninvolved free light chain ratio; presence of end organ damage (hypercalcemia, renal insufficiency, anaemia, bone involvement); International Staging System (ISS); glomerular filtration rate calculated from the updated equation of the Chronic Kidney Disease Epidemiology Collaboration (CKD-EPI) 2021 [31], and translocation and copy number events. Adverse cytogenetic abnormalities included the presence of t(4;14), t(14;16), t(14;20), del(17p) or gain(1q). Cytogenetic risk strata were defined as standard-risk (absence of any adverse cytogenetic abnormality), high-risk (1 adverse cytogenetic abnormality) and ultra-high-risk (>1 adverse cytogenetic abnormality) [32, 33].

### Statistical analysis

We evaluated the association of anthropometric variables with MM risk overall and stratified by race and sex. Among cases, we evaluated the association of BMI with the presence of MDE. We estimated the risk of MM using odds ratios (OR) and corresponding 95% confidence intervals (CI) calculated from logistic regression models adjusted for confounders including level of education (≤high school graduate; vocational, technical training or some college; ≥college graduate) and, for analyses not stratified by these variables, self-reported race (Black, White), sex and age (continuous, <55 years). Other potential confounders were evaluated but were excluded from final models because they were not related to MM risk or the anthropometric trait evaluated. BMI variables were categorised according to the World Health Organization (WHO) as underweight (<18.5 kg/m^2^), normal weight (reference category, 18.5–24.9 kg/m^2^), overweight (25.0–29.9 kg/m^2^), obese class I (30.0–34.9 kg/m^2^), obese class II (35.0–39.9 kg/m^2^) and obese class III (≥40.0 kg/m^2^). The small number of underweight participants (*n* = 12 cases; *n* = 14 controls) were excluded to minimise the influence of factors associated with acute pre-diagnostic weight loss on MM risk, consistent with a previously published report [34]. For cases, we calculated change (gain or loss) in height, weight, or BMI as the difference between those measures 1 year before diagnosis (usual adult) and at diagnosis (current). We modelled change in anthropometric variables (≥10% loss, <10% loss, no change [reference], <10% gain, ≥10% gain) mutually adjusted for continuous usual adult height, and/or weight and/or BMI (normal, overweight, obese I/II/III) as appropriate. A gain or loss of ≥10% was considered clinically significant [35]. Linear tests for trend were calculated using multivariable logistic regression with medians of anthropometric variable categories assigned to ordinal variables and modelled as continuous. Statistical significance was calculated using the maximum likelihood *χ*² test with multivariable model parameters, and differences between sex and race strata were determined using interaction terms. A two-sided *p* value of ≤0.05 was considered statistically significant. All analyses were conducted using SAS version 9.4 (Cary, NC).

## Results

### Demographics

Controls were similar to cases by race (Table 1). However, differences were observed by age and sex, despite frequency matching on these factors, due to a disproportionately higher participation rate among controls who were Black women (60%). The 849 MM cases were 54% men and had a median age of 62 years at diagnosis. Cases who self-identified as Black were significantly younger than those who self-identified as White (median age, 59 vs. 63 years; *p* < 0.0001) and reported less education (*p* < 0.0001) and lower annual household income at enrollment (*p* < 0.0001) than White cases.

**Table 1:** Select demographic characteristics of participants enrolled in the Integrative Molecular And Genetic Epidemiology (IMAGE) study, overall and stratified by race.

Demographic characteristic, *n* (%)^a^	Total population				Black participants			White participants		
	Total	Case	Control	*p*	Case	Control	*p*	Case	Control	*p*
	(*n* = 2176)	(*n* = 849)	(*n* = 1327)		(*n* = 342)	(*n* = 529)		(*n* = 507)	(*n* = 798)	
Male sex	982 (45.13)	455 (53.59)	527 (39.71)	<0.0001	152 (44.44)	174 (32.89)	0.001	303 (59.76)	353 (44.24)	<0.0001
Age, years										
Age, median (range)	63 (21–91)	62 (27–91)	64 (21–90)	<0.0001	59 (27–91)	61 (21–85)	0.003	63 (32–88)	66 (23–90)	0.0002
Age category										
≤40	68 (3.13)	27 (3.18)	41 (3.09)	<0.0001	17 (4.97)	24 (4.54)	0.01	10 (1.97)	17 (2.13)	0.001
41–50	208 (9.56)	104 (12.25)	104 (7.84)		61 (17.84)	54 (10.21)		43 (8.48)	50 (6.27)	
51–60	621 (28.54)	260 (30.62)	361 (27.20)		115 (33.63)	179 (33.84)		145 (28.60)	182 (22.81)	
61–70	845 (38.83)	328 (38.63)	517 (38.96)		113 (33.04)	197 (37.24)		215 (42.41)	320 (40.10)	
71–80	383 (17.60)	112 (13.19)	271 (20.42)		31 (9.06)	71 (13.42)		81 (15.98)	200 (25.06)	
≥81	51 (2.34)	18 (2.12)	33 (2.49)		5 (1.46)	4 (0.76)		13 (2.56)	29 (3.63)	
Education level										
High school graduate or less	541 (24.90)	308 (36.36)	233 (17.57)	<0.0001	157 (45.91)	124 (23.48)	<0.0001	151 (29.90)	109 (13.66)	<0.0001
Vocational, technical training or some college	748 (34.42)	271 (32.00)	477 (35.97)		104 (30.41)	243 (46.02)		167 (33.07)	234 (29.32)	
College graduate or post-graduate	884 (40.68)	268 (31.64)	616 (46.46)		81 (23.68)	161 (30.49)		187 (37.03)	455 (57.02)	
Smoking status^b^										
Nonsmoker	1248 (57.46)	490 (57.85)	758 (57.21)	0.77	204 (59.65)	296 (56.17)	0.31	286 (56.63)	462 (57.89)	0.65
Ever smoker	924 (42.54)	357 (42.15)	567 (42.79)		138 (40.35)	231 (43.83)		221 (43.37)	336 (42.11)	
Alcohol consumption^c^										
Nondrinker	1219 (56.12)	510 (60.28)	709 (53.47)	0.002	221 (64.62)	326 (61.74)	0.39	289 (57.34)	383 (47.99)	0.001
Ever drinker	953 (43.88)	336 (39.72)	617 (46.53)		121 (35.38)	202 (38.26)		215 (42.66)	415 (52.01)	
Annual household income										
≤$49,999	801 (40.80)	313 (42.88)	488 (39.58)	0.02	180 (61.02)	280 (57.85)	0.32	133 (30.57)	208 (27.77)	0.07
$50,000–$99,999	668 (34.03)	259 (35.48)	409 (33.17)		88 (29.83)	143 (29.55)		171 (39.31)	266 (35.51)	
≥$100,000	494 (25.17)	158 (21.64)	336 (27.25)		27 (9.15)	61 (12.60)		131 (30.11)	275 (36.72)	

^a^Percents may not add to 100 due to missing values.

^b^Smoking status (ever smoker) defined as smoked more than 100 cigarettes in a lifetime.

^c^Alcohol consumption (ever drinker) defined as at least one alcoholic beverage (beer, wine, hard liquor) per week for 6 months or longer.

### BMI and MM

Among controls, obesity was more common in Black participants, particularly among Black women (usual adult BMI ≥ 30.0 kg/m^2^: men, 35.29%; women, 61.54%) compared to White participants (men, 31.59%; women, 22.38%; *p*_interaction by race and sex_ = 0.02; Supplementary Table 1), consistent with the Southeastern US population [36]. Overall, we observed an 18% and a 14% increase in MM risk for every 5-kg/m^2^ increase in usual adult and current BMI, respectively (Table 2). Participants with a usual adult BMI in all three WHO obesity classes (≥30.0 kg/m^2^) had significant >65% increases in MM risk and those with a usual adult BMI ≥ 40 kg/m^2^ had the highest risk (OR = 1.87, 95% CI 1.25–2.80) compared to those with a normal usual adult BMI.

**Table 2:** Estimated risk of multiple myeloma associated with usual adult and current body mass index, overall and stratified by race and sex.

	**Total participants**^**a**^		**Black participants**				**White participants**				***p***_**interaction by race**_
	**OR (95% CI)**^**b**^	***p***	**Case (*n*** **= 338)**	**Control (*n*** **= 528)**	**OR (95% CI)**^**c**^	***p***	**Case (*n*** **= 499)**	**Control (*n* = 785)**	**OR (95% CI)**^**c**^	***p***	
Usual adult											
Body mass index (5 kg/m^2^)^d^	1.18 (1.09–1.27)	<0.0001	30.75 (19.72–75.52)	30.34 (18.88–68.22)	1.06 (0.95–1.18)	0.32	28.12 (18.83–74.73)	26.48 (18.56–50.49)	1.26 (1.13–1.40)	<0.0001	0.02
18.5–24.9 normal weight	1.00 (Reference)		36 (11.25)	81 (15.94)	1.00 (Reference)		114 (23.55)	270 (35.71)	1.00 (Reference)		
25.0–29.9 overweight	1.51 (1.17–1.94)	0.001	106 (33.13)	159 (31.30)	1.58 (0.98–2.56)	0.06	194 (40.08)	285 (37.70)	1.41 (1.04–1.90)	0.03	0.71
30–34.9 obese class I	1.67 (1.26–2.21)	0.0003	95 (29.69)	131 (25.79)	1.70 (1.04–2.78)	0.03	101 (20.87)	130 (17.20)	1.48 (1.04–2.12)	0.03	0.66
35.0–39.9 obese class II	1.68 (1.19–2.37)	0.003	46 (14.38)	84 (16.54)	1.32 (0.76–2.30)	0.32	50 (10.33)	44 (5.82)	2.05 (1.27–3.32)	0.003	0.25
≥40.0 obese class III	1.87 (1.25–2.80)	0.002	37 (11.56)	53 (10.43)	1.71 (0.93–3.15)	0.09	25 (5.17)	27 (3.57)	1.86 (1.01–3.42)	0.05	0.87
* p*-trend		0.0003				0.27				0.001	0.19
Current											
Body mass index (5 kg/m^2^)^e^	1.14 (1.07–1.23)	0.0002	30.56 (19.05–72.63)	30.51 (18.88–68.22)	1.01 (0.91–1.12)	0.88	28.30 (18.83–61.66)	26.67 (18.56–51.37)	1.24 (1.13–1.37)	<0.0001	0.004
18.5–24.9 normal weight	1.00 (Reference)		45 (13.72)	77 (15.40)	1.00 (Reference)		115 (23.28)	243 (32.66)	1.00 (Reference)		
25.0–29.9 overweight	1.26 (0.98–1.62)	0.07	113 (34.45)	156 (31.20)	1.32 (0.84–2.09)	0.24	178 (36.03)	287 (38.58)	1.15 (0.85–1.56)	0.37	0.62
30–34.9 obese class I	1.46 (1.11–1.93)	0.01	86 (26.22)	127 (25.40)	1.32 (0.82–2.13)	0.26	111 (22.47)	134 (18.01)	1.42 (0.99–2.02)	0.05	0.82
35.0–39.9 obese class II	1.33 (0.95–1.87)	0.10	48 (14.63)	86 (17.20)	1.02 (0.60–1.75)	0.93	50 (10.12)	52 (6.99)	1.54 (0.96–2.46)	0.07	0.25
≥40.0 obese class III	1.85 (1.26–2.72)	0.002	36 (10.98)	54 (10.80)	1.26 (0.69–2.30)	0.45	40 (8.10)	28 (3.76)	2.57 (1.48–4.46)	0.001	0.09
* p*-trend		0.001				0.92				0.0003	0.02
	**Total males**^**a**^		**Black males**				**White males**				***p***_**interaction by race**_
	**OR (95% CI)**^**f**^	***p***	**Case (*n*** **=** **150)**	**Control (*n*** **=** **173)**	**OR (95% CI)**^**g**^	***p***	**Case (*n*** **=** **302)**	**Control (*n*** **=** **353)**	**OR (95% CI)**^**g**^	***p***	
Usual adult											
Body mass index (5 kg/m^2^)	1.25 (1.11–1.42)	0.0004	30.41 (19.72–50.21)	28.66 (19.37–50.25)	1.32 (1.05–1.66)	0.02	28.13 (20.58–53.09)	27.37 (18.56–48.82)	1.22 (1.05–1.42)	0.01	0.61
18.5–24.9 normal weight	1.00 (Reference)		17 (12.06)	38 (22.35)	1.00 (Reference)		54 (18.31)	87 (25.22)	1.00 (Reference)		
25.0–29.9 overweight	1.52 (1.06–2.18)	0.02	50 (35.46)	72 (42.35)	1.73 (0.86-3.48)	0.13	134 (45.42)	149 (43.19)	1.44 (0.95–2.20)	0.09	0.69
30–34.9 obese class I	1.79 (1.20–2.68)	0.005	50 (35.46)	36 (21.18)	3.43 (1.63–7.22)	0.001	62 (21.02)	73 (21.16)	1.25 (0.76–2.04)	0.38	0.03
35.0–39.9 obese class II	1.77 (1.06–2.97)	0.03	19 (13.48)	20 (11.76)	1.95 (0.81–4.70)	0.14	30 (10.17)	24 (6.96)	1.80 (0.94–3.45)	0.08	0.90
≥40.0 obese class III	2.22 (1.06–4.64)	0.03	5 (3.55)	4 (2.35)	3.94 (0.90–17.36)	0.07	15 (5.08)	12 (3.48)	1.82 (0.77–4.26)	0.17	0.43
* p*-trend		0.004				0.01				0.10	0.22
Current											
Body mass index (5 kg/m^2^)	1.19 (1.06–1.34)	0.003	29.91 (19.05–55.53)	28.70 (19.37–50.25)	1.25 (1.01–1.53)	0.04	28.16 (19.73–53.56)	27.67 (18.56–50.18)	1.17 (1.01–1.35)	0.04	0.68
18.5–24.9 normal weight	1.00 (Reference)		27 (18.24)	37 (21.89)	1.00 (Reference)		57 (19.00)	78 (23.01)	1.00 (Reference)		
25.0–29.9 overweight	1.15 (0.80–1.63)	0.45	48 (32.43)	71 (42.01)	1.01 (0.53–1.92)	0.97	122 (40.67)	144 (42.48)	1.20 (0.78–1.84)	0.41	0.67
30–34.9 obese class I	1.43 (0.97–2.12)	0.07	46 (31.08)	35 (20.71)	2.06 (1.03–4.11)	0.04	70 (23.33)	78 (23.01)	1.17 (0.72–1.89)	0.52	0.20
35.0–39.9 obese class II	1.22 (0.74–2.02)	0.44	19 (12.84)	22 (13.02)	1.11 (0.49–2.52)	0.80	29 (9.67)	26 (7.67)	1.31 (0.69–2.50)	0.42	0.72
≥40.0 obese class III	2.44 (1.24–4.79)	0.01	8 (5.41)	4 (2.37)	3.17 (0.83–12.18)	0.09	22 (7.33)	13 (3.83)	2.20 (1.00–4.85)	0.05	0.68
* p*-trend		0.01				0.06				0.09	0.61
	**Total females**^**a**^		**Black females**				**White females**				***p***_**interaction by race**_
	**OR (95% CI)**^**f**^	***p***	**Case (*n*** **=** **188)**	**Control (*n*** **=** **355)**	**OR (95% CI)**^**g**^	***p***	**Case (*n*** **=** **197)**	**Control (*n*** **=** **432)**	**OR (95% CI)**^**g**^	***p***	
Usual adult											
Body mass index (5 kg/m^2^)	1.11 (1.01–1.22)	0.03	31.09 (21.03–75.52)	31.95 (18.88–68.22)	0.98 (0.86–1.11)	0.73	28.08 (18.83–74.73)	25.50 (18.61–50.49)	1.29 (1.11–1.49)	0.001	0.002
18.5–24.9 normal weight	1.00 (Reference)		19 (10.61)	43 (12.72)	1.00 (Reference)		60 (31.75)	183 (44.53)	1.00 (Reference)		
25.0–29.9 overweight	1.47 (1.03–2.10)	0.03	56 (31.28)	87 (25.74)	1.44 (0.75–2.77)	0.27	60 (31.75)	136 (33.09)	1.29 (0.83–2.00)	0.25	0.76
30–34.9 obese class I	1.46 (0.98–2.17)	0.06	45 (25.14)	95 (28.11)	0.96 (0.49–1.87)	0.90	39 (20.63)	57 (13.87)	1.80 (1.06–3.05)	0.03	0.13
35.0–39.9 obese class II	1.54 (0.96–2.46)	0.07	27 (15.08)	64 (18.93)	0.93 (0.45–1.91)	0.84	20 (10.58)	20 (4.87)	2.45 (1.19–5.01)	0.01	0.06
≥40.0 obese class III	1.50 (0.90–2.51)	0.12	32 (17.88)	49 (14.50)	1.16 (0.56–2.41)	0.70	10 (5.29)	15 (3.65)	1.85 (0.76–4.46)	0.17	0.30
* p*-trend		0.08				0.57				0.004	0.01
Current											
Body mass index (5 kg/m^2^)	1.10 (1.00–1.20)	0.05	30.85 (20.31–72.63)	32.45 (18.88–68.22)	0.93 (0.81–1.05)	0.23	28.34 (18.83–61.66)	25.84 (18.61–51.37)	1.31 (1.14–1.50)	0.0001	<0.0001
18.5–24.9 normal weight	1.00 (Reference)		18 (10.00)	40 (12.08)	1.00 (Reference)		58 (29.90)	165 (40.74)	1.00 (Reference)		
25.0–29.9 overweight	1.35 (0.94–1.93)	0.10	65 (36.11)	85 (25.68)	1.63 (0.84–3.17)	0.15	56 (28.87)	143 (35.31)	0.99 (0.63–1.55)	0.97	0.25
30–34.9 obese class I	1.40 (0.93–2.09)	0.10	40 (22.22)	92 (27.79)	0.94 (0.47–1.87)	0.86	41 (21.13)	56 (13.83)	1.81 (1.07–3.06)	0.03	0.13
35.0–39.9 obese class II	1.39 (0.87–2.21)	0.16	29 (16.11)	64 (19.34)	0.95 (0.46–1.98)	0.90	21 (10.82)	26 (6.42)	1.85 (0.94–3.66)	0.08	0.17
≥40.0 obese class III	1.49 (0.91–2.46)	0.12	28 (15.56)	50 (15.11)	1.01 (0.47–2.14)	0.99	18 (9.28)	15 (3.70)	2.79 (1.28–6.08)	0.01	0.03
* p*-trend		0.11				0.20				0.001	0.0003

*OR* odds ratio, *CI* confidence interval.

^a^Totals include cases and controls from the corresponding race and sex strata. Underweight individuals (12 cases and 14 controls) were excluded. Percents may not add to 100 due to missing values.

^b^Model adjusted for age, sex, education level and race.

^c^Model adjusted for age, sex and education level.

^d^Calculated from maximum adult height and weight from 1 year before diagnosis for cases; calculated from maximum adult height and from weight at enrollment for controls.

^e^Calculated from height and weight at diagnosis for cases; calculated from height and weight at enrollment for controls.

^f^Model adjusted for age, education level and race.

^g^Model adjusted for age and education level.

Considering usual adult BMI in sex-stratified analyses, the risk of MM was significantly elevated for both men and women (per 5 kg/m^2^ increase, OR = 1.25, 95% CI 1.11–1.42 and OR = 1.11, 95% CI 1.01–1.22, respectively; Table 2). When stratified jointly by sex and race, the magnitude of this association differed significantly across strata (*p*_interaction_ = 0.04; Supplementary Table 1) and was greatest for Black men (32% increase per 5 kg/m^2^ increase in usual adult BMI; *p* = 0.02). Likewise, Black men with a usual adult BMI ≥ 40 kg/m^2^ had the strongest increase in MM risk compared to those with a normal usual adult BMI (OR = 3.94, 95% CI 0.90–17.36). White men and women with a usual adult BMI ≥ 40 kg/m^2^ had similar, non-significant ~80% increases MM risk, whereas we observed no notable association among Black women in the highest usual adult BMI category compared to those with normal BMI (OR = 1.16, 95% CI 0.56–2.41). We observed similar associations with MM risk for current and usual adult BMI, except that the association of current BMI ≥ 40 kg/m^2^ with MM risk was strongest for White women compared to women with current normal BMI (*p* = 0.01). In case-case analyses stratified by age at diagnosis, Black patients with early age of onset (≤55 years) were 6-fold more likely to have obesity than White patients (OR = 6.19, 95% CI 2.46–15.56), although the difference by age strata was not statistically significant (*p* = 0.10; Supplementary Table 2).

### Weight and height and MM

Associations of weight with MM risk were directionally similar to BMI (Supplementary Table 1). Analyses stratified jointly by sex and race yielded increased MM risk estimates, particularly for persons in the heaviest usual weight quartile (Q4) compared to the lowest weight quartile (Q1) with similar magnitude for Black men (OR = 2.55, 95% CI 1.27–5.09), White men (OR = 2.66, 95% CI 1.61–4.40) and White women (OR = 2.73, 95% CI 1.56–4.78) but not for Black women (OR = 0.80, 95% CI 0.42–1.53). Associations observed for current weight were similar to those for usual adult weight. Considering maximum adult height, we noted a marginally significant trend of increased MM risk with increasing maximum adult height (*p*-trend = 0.07) that corresponded to a 28% elevated risk for patients in the tallest quartile compared to the shortest quartile (OR = 1.28, 95% CI 0.99–1.67; Supplementary Table 1). Current adult height was not notably associated with MM risk overall or in any participant subgroup.

We evaluated whether skeletal destruction contributed to loss in height among cases 1 year before diagnosis (Table 3). White MM patients showed significant reductions in height compared to Black MM patients, overall and within sex-specific strata (*p* ≤ 0.001). Nearly 80% of Black men and women, but only 53% of White men and women, presented at diagnosis with no change from maximum adult height. Only one White woman presented with a clinically significant reduction in height (≥10%), prohibiting further analysis of that degree of height loss. Of the remaining patients, we observed that White patients were approximately 3 times more likely than Black patients to show a clinically non-significant reduction in height at diagnosis independent of maximum adult height (White men, OR = 2.60, 95% CI 1.60–4.23; White women, OR = 3.20, 2.01–5.08). Additionally, among patients with a reduction in height, being overweight/obese the year before diagnosis (BMI ≥ 25 kg/m^2^) was associated with a non-significant 1.58-fold increased likelihood of presenting with lytic bone lesions (*p* = 0.33; Supplementary Table 3). However, in White women, being overweight/obese was strongly correlated with the presence of bony or extramedullary plasmacytoma (OR = 6.26, 95% CI 1.26–31.2), of which 66% were present in the spine. Regarding weight, Black participants were more likely than White participants to experience fluctuations in body weight in the year leading to diagnosis, especially among women (Table 3). Black women experienced clinically significant pre-diagnosis weight loss (*p* = 0.01) as well as non-significant weight loss (*p* = 0.003) and gain (*p* = 0.03), whereas White women maintained body weight during this same timeframe. Consistent with racial differences in height loss at diagnosis, White patients were more likely than Black patients to experience an increase in BMI in the year before diagnosis independent of maximum adult BMI (*p* ≥ 0.01).

**Table 3:** Change in usual adult and current height, weight and body mass index in the year before diagnosis among Black and White patients with multiple myeloma, overall and stratified by sex.

	**Total cases**^**a**^				**Males**^**a**^				**Females**^**a**^				***p***_**interaction by sex and race**_
	**Black cases (*n* = 338)**	**White cases (*n* = 499)**	**OR (95% CI)**^**b**^	***p***	**Black cases (*n* = 150)**	**White cases (*n*** **= 302)**	**OR (95% CI)**^**b**^	***p***	**Black cases (*n*** **= 188)**	**White cases (*n*** **= 197)**	**OR (95% CI)**^**b**^	***p***	
Change in height, mean (±sd) cm^c^	0.86 (2.10)	2.18 (3.29)	1.20 (1.12–1.28)	<0.0001	0.80 (2.04)	2.08 (3.11)	1.20 (1.08–1.33)	0.001	0.91 (2.15)	2.35 (3.55)	1.20 (1.09–1.31)	0.0001	0.97
Clinically significant change in height, *n* (%)													
No change	267 (79.46)	265 (53.32)	1.00 (Reference)		120 (80.00)	164 (54.49)	1.00 (Reference)		147 (79.03)	101 (51.53)	1.00 (Reference)		
<10% loss	69 (20.54)	231 (46.48)	2.88 (2.07–4.01)	<0.0001	30 (20.00)	137 (45.51)	2.60 (1.60–4.23)	0.0001	39 (20.97)	94 (47.96)	3.20 (2.01–5.08)	<0.0001	0.57
≥10% loss	0 (0.00)	1 (0.20)	ND		0 (0.00)	0 (0.00)	ND		0 (0.00)	1 (0.51)	ND		
Change in weight, mean (±sd) kg^d^	2.23 (9.54)	1.29 (7.20)	0.99 (0.97–1.01)	0.28	2.01 (10.26)	1.76 (7.29)	1.00 (0.97–1.02)	0.90	2.41 (8.94)	0.55 (7.00)	0.98 (0.95–1.01)	0.19	0.20
Clinically significant change in weight, *n* (%)													
≥10% loss	48 (15.19)	52 (10.77)	0.54 (0.34–0.87)	0.01	16 (11.43)	34 (11.53)	0.90 (0.43–1.87)	0.78	32 (18.18)	18 (9.57)	0.38 (0.19–0.78)	0.01	0.06
<10% loss	89 (28.16)	94 (19.46)	0.51 (0.34–0.74)	0.001	46 (32.86)	63 (21.36)	0.61 (0.36–1.04)	0.07	43 (24.43)	31 (16.49)	0.40 (0.22–0.73)	0.003	0.26
No change	110 (34.81)	229 (47.41)	1.00 (Reference)		56 (40.00)	135 (45.76)	1.00 (Reference)		54 (30.68)	94 (50.00)	1.00 (Reference)		
<10% gain	53 (16.77)	85 (17.60)	0.82 (0.54–1.26)	0.37	16 (11.43)	51 (17.29)	1.71 (0.86–3.42)	0.13	37 (21.02)	34 (18.09)	0.51 (0.28–0.93)	0.03	0.01
≥10% gain	16 (5.06)	23 (4.76)	0.79 (0.39–1.59)	0.50	6 (4.29)	12 (4.07)	1.19 (0.40–3.58)	0.75	10 (5.68)	11 (5.85)	0.68 (0.26–1.76)	0.43	0.54
Change in BMI, mean (±sd) kg/m^2 e^	0.46 (3.31)	−0.36 (2.54)	0.93 (0.88–0.98)	0.01	0.37 (3.30)	−0.15 (2.34)	0.96 (0.88–1.04)	0.29	0.53 (3.33)	−0.70 (2.79)	0.89 (0.82–0.96)	0.004	0.16
Clinically significant change in BMI, *n* (%)													
≥10% loss	43 (13.65)	32 (6.64)	0.88 (0.41–1.89)	0.74	16 (11.43)	23 (7.80)	1.22 (0.44–3.43)	0.70	27 (15.43)	9 (4.81)	0.68 (0.18–2.49)	0.56	0.44
<10% loss	130 (41.27)	146 (30.29)	1.10 (0.58–2.10)	0.78	64 (45.71)	86 (29.15)	1.07 (0.46–2.47)	0.88	66 (37.71)	60 (32.09)	1.33 (0.44–4.00)	0.62	0.77
No change	23 (7.30)	25 (5.19)	1.00 (Reference)		14 (10.00)	18 (6.10)	1.00 (Reference)		9 (5.14)	7 (3.74)	1.00 (Reference)		
<10% gain	97 (30.79)	235 (48.76)	2.19 (1.15–4.16)	0.02	38 (27.14)	150 (50.85)	2.90 (1.24–6.75)	0.01	59 (33.71)	85 (45.45)	1.86 (0.62–5.56)	0.27	0.54
≥10% gain	22 (6.98)	44 (9.13)	2.00 (0.90–4.45)	0.09	8 (5.71)	18 (6.10)	1.87 (0.58–5.99)	0.29	14 (8.00)	26 (13.90)	2.97 (0.86–10.27)	0.09	0.54
Magnitude percent change in BMI, *n* (%)													
No change	23 (7.30)	25 (5.19)	1.00 (Reference)		14 (10.00)	18 (6.10)	1.00 (Reference)		9 (5.14)	7 (3.74)	1.00 (Reference)		
<10% change	227 (72.06)	381 (79.05)	1.58 (0.85–2.95)	0.15	102 (72.86)	236 (80.00)	1.79 (0.80–3.99)	0.15	125 (71.43)	145 (77.54)	1.59 (0.55–4.66)	0.40	0.88
≥10% change	65 (20.63)	76 (15.77)	1.29 (0.65–2.59)	0.47	24 (17.14)	41 (13.90)	1.47 (0.57–3.74)	0.42	41 (23.43)	35 (18.72)	1.56 (0.49–4.91)	0.45	0.93

*OR* odds ratio, *CI* confidence interval, *BMI* body mass index.

^a^Percents may not add to 100 due to missing values.

^b^Risk estimates adjusted for age at diagnosis, education level and usual adult height, weight or usual adult BMI (normal, overweight, obese), as appropriate. Risk estimates were calculated using Black patients as the reference group.

^c^Change in height calculated from current height (at diagnosis) subtracted from usual adult height (1 year before diagnosis).

^d^Change in weight calculated from current weight subtracted from usual adult weight.

^e^Change in BMI calculated from current BMI subtracted from usual adult BMI.

### Myeloma-defining events and BMI

In case only analysis, distributions of diagnostic MDE differed among MM patients with normal compared to overweight/obese BMI (Table 4). Overall, relative to patients with normal BMI, those with overweight/obesity were more likely to present with low renal function (CKD-EPI eGFR ≤60; OR = 1.62, 95% CI 1.09–2.40), deletion 13q (OR = 1.73, 95% CI 1.08–2.76) and lytic lesions or compression fractures (OR = 2.39, 95% CI 0.82–7.01) and less likely to present with severe diffuse osteopenia (OR = 0.51, 95% CI 0.31–0.81) and lytic lesions located in the spine (OR = 0.47, 95% CI 0.26–0.87); they also had a suggestive non-significant increase in ISS stage (ISS-III: OR = 1.36, 95% CI 0.79–2.37). Among jointly defined sex- and race- strata, overweight/obese Black men were notably less likely than Black men with normal weight to present with serum monoclonal protein ≥3 g/dl (OR = 0.19, 95% CI 0.04–0.97) and non-IgG isotype (OR = 0.20, 95% CI 0.06–0.74), whereas overweight/obese White men were less likely than White men with normal weight to present with the reciprocal translocation of the IGH locus involving *FGFR3* (t(4;14); OR = 0.14, 95% CI 0.05–0.46) and severe diffuse osteopenia (OR = 0.36, 95% CI 0.16–0.81; Supplementary Table 4). Likewise, overweight/obese White women were less likely than White women with normal weight to present with light chain restricted disease (OR = 0.49, 95% CI 0.25–0.99).

**Table 4:** Presence of diagnostic clinical features among multiple myeloma patients with overweight and obesity.

Clinical feature, *n* (%)^a^	Normal BMI (*n* = 150)	BMI ≥ 25.0 (*n* = 654)	OR (95% CI)^b^	*p*
Tumour burden				
Bone marrow plasma cells ≥60%	64 (44.76)	274 (44.26)	0.95 (0.66–1.38)	0.80
β2-microglobulin, median (range) mg/l	3.40 (1.30–21.00)	3.81 (0.23–91.40)	1.34 (0.66–2.71)	0.42
Lactate dehydrogenase ≥240 units	13 (8.67)	62 (9.48)	1.02 (0.54–1.95)	0.94
GFR ≤ 60, *n* (%)^c^	47 (31.33)	270 (41.28)	1.62 (1.09–2.40)	0.02
International staging system				
I	38 (36.19)	139 (30.28)	1.00 (Reference)	
II	37 (35.24)	172 (37.47)	1.30 (0.77–2.18)	0.64
III	30 (28.57)	148 (32.24)	1.36 (0.79–2.37)	0.47
Paraprotein assessment^d^				
Serum monoclonal protein, ≥3 g/dl	47 (44.34)	231 (45.03)	0.97 (0.63–1.49)	0.89
Non-IgG isotype	32 (28.83)	129 (26.22)	0.93 (0.58–1.48)	0.76
Light chain restricted disease	34 (23.13)	127 (20.06)	0.85 (0.55–1.32)	0.47
Kappa	94 (64.83)	396 (65.78)	1.02 (0.69–1.51)	0.92
Lambda	51 (35.17)	206 (34.22)	0.98 (0.66–1.45)	0.92
Involved:uninvolved serum FLC ratio ≥100	48 (42.86)	208 (41.94)	1.01 (0.66–1.55)	0.95
Chromosomal abnormality				
IGH translocation, any	28 (38.89)	115 (34.33)	0.82 (0.48–1.41)	0.47
t(4;14) (*FGFR3*)	9 (12.50)	24 (7.10)	0.54 (0.23–1.25)	0.15
t(14;16) (*MAF*)	4 (5.80)	23 (7.28)	1.48 (0.47–4.64)	0.50
t(11;14) *(CCND1)*	16 (21.33)	70 (19.23)	0.83 (0.44–1.56)	0.56
del(13q)	38 (37.25)	212 (44.17)	1.73 (1.08–2.76)	0.02
del(17p) (*TP53*)	13 (19.12)	62 (18.62)	1.02 (0.52–2.03)	0.94
Amplification chr 1q	24 (35.82)	125 (41.53)	1.43 (0.80–2.54)	0.22
Cytogenetic risk^e^				
SR	55 (59.14)	270 (60.00)	1.00 (Reference)	
HiR	29 (31.18)	128 (28.44)	0.95 (0.57–1.58)	0.46
UHiR	9 (9.68)	52 (11.56)	1.38 (0.63–3.03)	0.37
End organ damage^f^				
Hypercalcemia	22 (17.05)	123 (21.06)	1.26 (0.76–2.11)	0.37
Renal insufficiency	23 (17.97)	144 (24.24)	1.46 (0.88–2.40)	0.14
Anaemia	71 (54.62)	331 (55.17)	0.92 (0.62–1.36)	0.66
Bone involvement	110 (79.71)	501 (81.20)	1.13 (0.70–1.82)	0.63
* p*-trend				0.22
Bone involvement				
Severe diffuse osteopenia	32 (29.36)	83 (16.94)	0.51 (0.31–0.81)	0.01
Lytic bone lesions	94 (86.24)	453 (91.70)	1.50 (0.78–2.88)	0.22
Compression fractures	48 (44.04)	177 (35.83)	0.71 (0.46–1.09)	0.11
Durie-Salmon Bone Scale				
Osteopenia only	6 (5.50)	11 (2.23)	1.00 (Reference)	
Lytic lesion(s) or compression fracture(s)	65 (59.63)	343 (69.43)	2.39 (0.82–7.01)	0.05
Lytic lesion(s) and compression fracture(s)	38 (34.86)	140 (28.34)	1.63 (0.54–4.90)	0.87
Lytic lesion count				
1–4	22 (25.00)	107 (25.36)	1.00 (Reference)	
5–10	28 (31.82)	140 (33.18)	0.97 (0.52–1.82)	0.82
11–20	20 (22.73)	99 (23.46)	1.09 (0.55–2.15)	0.47
>20	18 (20.45)	76 (18.01)	0.71 (0.35–1.45)	0.23
* p*-trend				0.49
Location of lytic lesion				
Appendicular skeleton	48 (64.00)	268 (69.97)	1.43 (0.84–2.44)	0.19
Axial skeleton	72 (94.74)	349 (89.49)	0.46 (0.16–1.35)	0.16
Spine	59 (78.67)	261 (67.44)	0.47 (0.26–0.87)	0.02

*BMI* body mass index, *OR* odds ratio, *CI* confidence interval, *GFR* glomerular filtration rate, *IgG* immunoglobulin (Ig)-G, *FLC* free light chain, *SR* standard risk, *HiR* high-risk, *UHiR* ultra-high-risk.

^a^Percents may not add to 100 due to missing values.

^b^Adjusted for age (continuous), education (high school graduate or less, vocational, technical training or some college, college graduate or post-graduate), race, and sex.

^c^GFR was calculated from the updated Chronic Kidney Disease Epidemiology Collaboration.

^d^Cases with biclonal disease were excluded (*n* = 10).

^e^Adverse cytogenetic risk strata: standard risk (SR) defined as presence of 0, high risk (HR) defined as presence of 1, and ultra-high risk (UHiR) defined as presence of ≥1 cytogenetic abnormality.

^f^More than 1 organ damage may be present in patients with MM. Therefore, percents may be greater than 100.

## Discussion

MM is the most common haematologic malignancy in Black populations. However, our understanding of MM aetiology is largely based on studies from patients who self-identify as White. This study expands prior investigations of obesity and MM risk, to elucidate the role of anthropometric traits in the excess risk of MM observed among Black participants, and the clinical presentation of disease.

Findings from our study support a similarly elevated risk of MM in persons with higher usual adult and current BMI, with slightly higher magnitudes of effect among men due to the high prevalence of obesity among controls who were Black women. Higher risk estimates were most apparent for participants with severe obesity (BMI ≥ 40 kg/m^2^), particularly among Black men, albeit based on a small sample. Among cases, Black patients were significantly heavier than White patients, and in the year before MM diagnosis, Black women were more likely to experience notable fluctuations in body weight, whereas White men and women showed marked reductions in height. These patterns contributed to pre-diagnosis fluctuations in BMI that may be a consequence of disease. In an evaluation of MDE, MM patients with overweight/obesity were more likely to present with low renal function, deletion 13q and lytic lesions or compression fractures, whereas severe diffuse osteopenia and lytic lesions occurring in the spine were less common, suggesting an aetiologic role for adiposity in underlying bone disease.

Obesity is a well-established modifiable risk factor for MM [26]. The overall positive association of elevated MM risk and BMI observed in our study is consistent with prior evidence from meta- and pooled-analyses of case-control and prospective cohort studies [8, 13, 18–21, 23, 37–40]. Our observation of an overall 18% increased risk of MM associated with each additional 5 kg/m^2^ in usual adult BMI is stronger than those previously reported from an International Multiple Myeloma Consortium pooled analysis of 2318 incident MM cases and 9609 controls (9% increase per additional 5 kg/m^2^) [19] and a recent pooled analysis of six US-based prospective cohort studies of 544,016 participants (10% increase per additional 5 kg/m^2^) [18], possibly reflecting the use by those studies of time-varying repeated measures for BMI that were unavailable in our study. However, our observation is similar in magnitude to an earlier pooled analysis of three cohorts, including two from the aforementioned six-cohort pooled analysis (17% increase per additional 5 kg/m^2^ BMI) [21]. Our observation for a slightly stronger association of usual adult BMI with MM risk in men than in women coincides with findings from some [13, 17, 21] but not all prior reports [18]. The elevated risk of MM associated with BMI ≥ 30 kg/m^2^ observed in our study is consistent with previous findings summarised from meta- and pooled-analyses yielding risk estimates ranging from 1.2- to 1.6-fold [8, 19], and we note a similar magnitude of effect with severe obesity (BMI ≥ 40 kg/m^2^) from a recent independent pooled analysis of prospective cohort studies (HR = 1.77) [18]. Existing evidence supports a positive association of both early and later adult BMI with MM risk [18–21], whereas persons with a lean and stable body shape trajectory demonstrate a reduction in MM risk [22]. Collectively, these findings suggest that cumulative exposure to adiposity over the lifespan is important in MM aetiology [18], which is further substantiated by evidence supporting a role for obesity in the progression of MGUS to MM [9–12].

Our study included a larger sample of Black participants than previously published studies, thereby facilitating detailed race- and sex-specific analyses that also included evaluations of severe obesity (BMI ≥ 40 kg/m^2^). In general, our findings from race-stratified analyses support evidence from a meta-analysis of three studies that showed a positive relationship between obesity and MM risk that was similar in magnitude among White (RR = 1.33, 95% CI 0.96–1.84) and Black participants (RR = 1.30, 95% CI, 1.08–1.58) [6], notwithstanding that obesity was defined as BMI ≥ 25.78 kg/m^2^, a range that included both overweight and obese individuals, in one of these studies [16]. However, in our sex- and race-stratified analysis, we noted a significant ~3-fold elevated risk of MM among obese Black men with a usual adult BMI ≥ 30 kg/m^2^ with significant differences across sex and race strata. Moreover, we note a stronger suggestive ~4-fold effect for Black men with severe obesity (≥40 kg/m^2^), which has not been previously reported. Our finding for Black men is noteworthy given the rising prevalence of severe obesity in the US, which has tripled in recent years from 2.8% in 1988 to 9.2% in 2018 [41], particularly among Black adults for whom both standardised incidence rates of MM and age-adjusted obesity (49.9%) are highest [42]. Thus, the excess risk of MM due to obesity may contribute to the increased incidence of MM observed in Black men. Alternatively, the increased risk of MM associated with obesity among Black persons could be influenced by underlying obesity loci, which are more frequent in this population [43]. Studies are warranted to examine these relationships.

Following BMI, findings for usual adult and current weight and MM risk are consistent with previous reports [18, 19, 23, 25, 44], which support a positive association of weight and MM risk at both time points, albeit with weaker associations than our observations. Similarly, the modest non-significant increased risk of MM observed among taller persons in our study is consistent with most [24, 25, 44–48] but not all prior reports of a positive relation between adult height and MM risk [15, 18, 23]. Our observation that height was notably reduced among White patients in the year leading to diagnosis has not been previously reported and suggests that height may be deleteriously influenced by the disease process, leading to an artificial inflation of BMI, particularly among White patients [49]. Thus, weight may be the more important contributor to body size underlying MM risk particularly during the transition from early to late-stage disease.

A unique strength of our study is the ability to evaluate the contribution of BMI to the presence of diagnostic MDE and differences by race. We hypothesised that MM patients with overweight/obesity are more likely to present with clinical features consistent with increased tumour burden. Although we noted a significant increase in the presence of lytic lesions or compression fractures, deletion 13q and a suggestive increase in ISS stage among MM patients with overweight/obesity overall, we did not observe differences in other clinical features associated with increased tumour burden. The lack of association may reflect inadequate statistical power to detect modest effects. Accordingly, we acknowledge the possibility that findings could be due to chance. Additional studies are required to confirm these findings and investigate a biological basis.

Although the mechanistic role of obesity in myelomagenesis is unclear, growing evidence supports a strong rationale. Adipose tissue is the largest secretory endocrine organ capable of dysregulating inflammation and perturbing endogenous hormone and metabolic pathways. Adipocytes secrete adipokines, which include proteins (leptin, resistin) and pro-inflammatory cytokines (interleukin [IL]-6, TNF-α) and anti-inflammatory or insulin-sensitising cytokines (adiponectin) that act in an autocrine and paracrine manner. In the bone marrow microenvironment, IL-6 is a driver of B cell hematopoiesis [50, 51] and the homing, survival and proliferation of monoclonal CD138+ bone marrow plasma cells through the downstream regulatory effects of anti-apoptotic transcription factors from NF-κB signal transduction pathways. In the periphery, adipokines can contribute to a chronic, low-grade, obesity-related inflammatory state, which leads to insulin resistance and hyperinsulinemia that indirectly contributes to myelomagenesis through the action of bioavailable insulin [52] on circulating endogenous growth factors (insulin-like growth factor [IGF]) [53], and their binding proteins [54]. Indeed, high levels of circulating IGF-1, IGF-binding protein-1, and soluble IL-6 receptor, as well as low adiponectin levels have been consistently associated with MM risk [55–58] and progression [58]. Causality is further substantiated from an experimental model demonstrating the induction of a MM-like condition in mice with diet-induced obesity [59]. Thus, adipokines could be important factors linking adiposity in the bone marrow microenvironment and periphery with chronic inflammation thereby promoting osteoclastogenesis and subsequent skeletal destruction. Future studies are necessary to delineate these complex relationships and their relevance to skeletal destruction and other MDE.

Our investigation was designed to evaluate risk factors associated with the excess risk of MM observed among Black participants using well-characterised self-reported Black and White MM cases and controls. However, the interpretation of our findings is not without limitation. Despite efforts to control for factors known to influence BMI and MM associations, frequency matching on sex and race was imprecise, lending the possibility of residual confounding. Moreover, we did not directly evaluate environmental, geographical or structural factors that could influence racialized groups or BMI, further contributing to the possibility of confounding. In addition, due to the high proportion of obesity, particularly among Black women, we may have underestimated the association of obesity with MM risk in Black participants, although it is possible that other factors may have contributed to an attenuation of risk. Furthermore, the high prevalence of obesity limited our ability to evaluate the influence of BMI on other meaningful MM risk strata (e.g., adverse cytogenetic abnormalities). However, the consistency of findings with prospective cohort studies suggest that any potential bias was minimal. Finally, although BMI is a practical metric to estimate body fatness for population studies, it is a poor estimate of body composition [60]. Therefore, other potentially relevant anthropometric traits such as repeated direct measures of body composition, including lean mass, fat mass and adiposity internal and external to the bone marrow microenvironment may be useful in further delineating the mechanistic role of obesity in myelomagenesis and subsequent clinicopathologic features.

In summary, we confirm a positive association of BMI with MM risk that was particularly strong for Black men and for the first time, describe variation in the presence of MDE and clinical features among MM patients who were overweight and obese. The consistency of our results supports obesity as a modifiable risk factor for MM and highlights the importance of improving our understanding of the underlying biological mechanisms by which obesity, internal and external to the bone marrow microenvironment, promotes myelomagenesis and affects the presentation of disease. Such characterisations will be valuable to enable the translation of these findings to effective energy balance interventions as a strategy to reduce MM risk, as well as the transition from early to late-stage disease, particularly in high-risk populations such as Black adults for whom obesity and MM are increasingly more common.

### Supplementary information

BMI and MM Risk List of Supplemental Tables

BMI and MM Risk Supplemental Table 1

BMI and MM Risk Supplemental Table 2

BMI and MM Risk Supplemental Table 3

BMI and MM Risk Supplemental Table 4

## Data Availability

Data described in the manuscript will not be made publicly available due to participant confidentiality and privacy concerns. Inquiries with reasonable requests for data access may be made to the corresponding author.
