# Supplementary material for: Anthropometric traits and risk of multiple myeloma: differences by race, sex and diagnostic clinical features
Source: Br J Cancer. 2024 Jun 7;131(2):312–24. doi: 10.1038/s41416-024-02723-6 (PMC11263363; doi:10.1038/s41416-024-02723-6)
Supplement: Supplementary file 1 — BMI and MM Risk List of Supplemental Tables [file 41416_2024_2723_MOESM1_ESM.docx]

**LIST OF SUPPLEMENTAL TABLES**

**Supplemental Table 1.** Estimated risk of multiple myeloma associated with usual adult and current body mass index and other anthropometric traits, overall and stratified by sex and race

**Supplemental Table 2.** Usual adult anthropometric traits and early age at diagnosis among Black and White patients with multiple myeloma

**Supplemental Table 3.** Presence of bone involvement among multiple myeloma patients with overweight and obesity and a reduction in height one year before diagnosis, overall and stratified by sex and race

**Supplemental Table 4.** Presence of diagnostic clinical features among multiple myeloma patients with overweight and obesity, overall and stratified by sex and race
